# Integrative oncology: evolution, framework, and future directions

**DOI:** 10.3389/fonc.2026.1838177

**Published:** 2026-06-05

**Authors:** Lorenzo Cohen, Eran Ben-Arye

**Affiliations:** 1 Department of Palliative, Rehabilitation, & Integrative Medicine, The University of Texas MD Anderson Cancer Center, Houston, TX, United States; 2 Integrative Oncology Program, The Oncology Service, Lin-Carmel-Zebulun Medical Centers, Rappaport Faculty of Medicine, Technion-Israel Institute of Technology, Haifa, Israel; 3 Middle East Research Group in Integrative Oncology (MERGIO), Within Middle East Cancer Consortium (MECC), Haifa, Israel; 4 Society for Integrative Oncology (SIO) Regional Ambassador to Europe and the Middle East, Haifa, Israel

**Keywords:** integrative medicine, lifestyle, oncology, quality of life, safety, survivorship

## Abstract

Over the past several decades, cancer treatment has evolved from a discipline centered almost exclusively on tumor-directed modalities to one that increasingly acknowledges the complex interplay of biological, behavioral, psychosocial, spiritual, cultural, and environmental factors that shape patient outcomes. This shift has set the stage for the rise of integrative oncology: an evidence-informed, patient-centered approach that combines mind–body practices, natural products, and lifestyle strategies with conventional cancer treatment to enhance wellbeing, manage symptoms, and improve quality of life across the cancer continuum. Although rooted in long-standing healing traditions, integrative oncology emerged as a modern clinical and scientific field in response to patient needs, health-belief models, growing research evidence, and recognition of the limitations of a purely biomedical framework. There is also a need to mitigate the risks associated with unmonitored “alternative medicine” use and to ensure that trained healthcare professionals foster open, non-judgmental, evidence-based, patient-centered discussions regarding the use of integrative modalities in high-risk oncology and surgical settings. The formation of the Society for Integrative Oncology, development of evidence-informed clinical guidelines, and increasing adoption within major cancer centers have collectively advanced the discipline and established standards for safe and effective care. As the field matures, challenges remain including disparities in access, gaps in education, and the need for rigorous whole-systems research. This introductory manuscript to the special issue outlines the historical trajectory, scientific foundations, clinical models, and future opportunities that define integrative oncology today and its potential to transform cancer care.

## Introduction

The field of oncology has undergone a profound transformation over the course of the past half-century, expanding from a primary focus on tumor-directed interventions to a more comprehensive understanding of what constitutes high-quality cancer care. Advances in molecular biology, genomics, targeted therapies, immunotherapy, and supportive care have significantly improved survival for many cancer types. Yet these achievements have also highlighted the limits of a strictly biomedical framework. Patients continue to experience substantial symptom burden, emotional distress, functional impairment, and social disruption throughout their cancer journey. This is especially salient today as cancer survival increases, leading to more people living with and beyond cancer than ever before. These realities have contributed to the emergence and rapid development of integrative oncology, a discipline that seeks to combine evidence-based complementary approaches within conventional oncologic treatment settings in a manner that enhances patient overall well-being, symptom control, and overall clinical outcomes, while aligning with the person’s health beliefs, culture, expectations, and unmet needs.

Although integrative oncology is a contemporary field, the concept that health arises from interactions among biological, psychological, spiritual, behavioral, cultural, and environmental factors is deeply rooted in the history of healing systems. Many ancient medical traditions emphasized the inseparability of mental and physical states, the role of lifestyle, and the importance of aligning clinical care with a person’s values, social relationships, and environment (1, 2). Western biomedicine, while achieving extraordinary success in understanding and targeting disease mechanisms, eventually oriented itself toward technological and pharmacologic solutions. By the twentieth century, the focus of oncology had become increasingly specialized, and many dimensions of the patient and the informal caregiver experience, including nutrition, stress, daily activity, sleep, emotional resilience, and concerns while facing existential fear of death and disability, were left largely outside the scope of routine care. Yet patients continued to seek meaning, agency, and holistic support (3), and clinicians increasingly recognized gaps in conventional cancer care, particularly around symptom burden, quality of life, and lifestyle determinants of outcomes.

These converging forces fueled the rise of a more comprehensive model of care, one that honors biological complexity and human experience in equal measure. The development of integrative oncology can therefore be viewed both as a response to these historical gaps in care and as a reflection of growing scientific evidence demonstrating the measurable clinical benefits of behavioral and complementary medicine interventions. The emergence of integrative oncology has occurred alongside the evolution of other holistic, patient-centered disciplines including family medicine (4), palliative care (5), and narrative medicine (6). There is also the increasing recognition by global authorities such as the World Health Organization of the importance of patients’ cultural perspectives, health beliefs, and use of traditional and herbal medicine (7).

Today, integrative oncology is an established component of comprehensive cancer care across many leading centers worldwide (8, 9). Evidence-based guidelines, robust clinical research, growing professional societies, and the engagement of major oncology organizations collectively signal the field’s maturation (10). This introduction offers a contemporary perspective on integrative oncology—examining its definition, historical trajectory, clinical models, research foundations, and future directions—while highlighting the opportunities that lie ahead for researchers, clinicians, and patients.

## Defining integrative oncology

As oncology practice evolved, so too did the terminology used to describe approaches outside the conventional biomedical paradigm. Early discussions framed these practices as *alternative*—implying a replacement for standard therapies—or *complementary*, referring to supportive therapies used adjunctively. Over time, such designations proved inadequate, both scientifically and clinically. They did not capture the intentional, evidence-informed approach that many clinicians sought, nor did they reflect the bidirectional exchange between biomedical and non-biomedical traditions and the need to establish communication among integrative practitioners, oncologists, radiologists, oncological surgeons, palliative care providers, oncology nurses, psycho-oncologists and other healthcare professionals collaborating within the interdisciplinary team.

The contemporary definition of integrative oncology comes from an international consensus (11) that characterized it as: “*patient-centered, evidence-informed approach to cancer care that utilizes mind–body practices, natural products, and lifestyle strategies from diverse traditions alongside conventional cancer treatments, with the goal of optimizing health, improving quality of life, and enhancing clinical outcomes across the cancer continuum”*. This definition emphasizes three essential elements: scientific rigor, safety, and whole-person care. Integrative oncology is not synonymous with unregulated complementary or alternative therapies, nor does it imply rejection of standard oncologic treatment. Rather, it denotes an intentional synthesis of approaches, each evaluated on its own merits, to address the full spectrum of patient needs across prevention, active treatment, survivorship, and end-of-life care.

Within this framework, lifestyle medicine plays a foundational role. A substantial body of research demonstrates that diet, physical activity, sleep, stress physiology, and social connection influence cancer incidence, treatment response, recurrence risk, and overall mortality (12). There is also evidence for the synergy of these factors, both in terms of the success related to behavior change as well as clinical outcomes. Although these factors are modifiable and clinically significant, they have historically been underemphasized in mainstream oncology practice. Integrative oncology explicitly incorporates them into clinical decision-making and individualized care planning.

From a global perspective, the emergence and implementation of integrative oncology is also intimately related to cross-cultural medicine and to the significant affinity of patients with traditional medicine modalities of care, such as herbal medicine, touch, and spiritual interventions prevalent worldwide, particularly in Asia, Africa, South and Central America, as well as among minority groups in affluent countries (13).

## Historical context and early development

The development of integrative oncology should be seen in the wider context of integrative medicine services, officially recognized during the first half of the 20^th^ century in Germany and the United Kingdom, and later in China, India, Switzerland, and other countries with an increasing emphasis on integrative cancer care (14). The establishment of integrative oncology within academic medical centers accelerated in the late 1990s and early 2000s, spurred in part by patient interest, clinician uncertainty regarding supplement and natural products safety, and a growing recognition that comprehensive supportive care was essential to improve quality of life and treatment adherence. Several major cancer centers established dedicated integrative oncology programs, creating environments where evidence-based complementary therapies could be delivered by trained professionals working in close partnership with oncology teams. These programs also generated platforms for rigorous research of acupuncture, yoga, meditation, massage, and other modalities. As clinical services expanded, it became increasingly clear that a coherent, interdisciplinary professional community was needed to advance research, education, and clinical standards.

## The formation of the Society for Integrative Oncology

The founding of the Society for Integrative Oncology (SIO) in 2003 represented a pivotal moment in the maturation of the field. The society brought together oncologists, nurses, psychologists, complementary therapy providers, nutrition professionals, health behavior researchers, and patient advocates with the shared goal of fostering scientific rigor, clinical safety, and interdisciplinary collaboration. In one of the early leadership conversations about the name of the society, we debated whether it should be the Society for Integrative Oncology or the Society *of* Integrative Oncology. The American Society of Clinical Oncology uses the term “of”, so that option made sense for us to align with ASCO, a future partner. Yet we agreed we wanted a society and community that stood “for” integrative oncology as a field, and so the name was codified.

The society’s early conferences drew hundreds of attendees and helped legitimize integrative oncology within conventional oncology. Over the years, SIO has partnered with major oncology organizations, expanded internationally, and developed authoritative clinical guidelines that continue to shape practice worldwide. The SIO has developed a mechanism for disseminating emerging evidence, cultivating methodological rigor, and articulating principles of best practice.

## Clinical guidelines

As the evidence base for integrative therapies grew, major oncology organizations began to incorporate these findings into guideline development. Early clinical practice guidelines focused on lung cancer (15) and emphasized the importance of informed dialogue between clinicians and patients regarding the use of complementary therapies. Recommendations highlighted the utility of acupuncture for nausea and pain, mind–body practices for mood and sleep disturbances, massage therapy for anxiety and pain, and exercise and dietary strategies for symptom management (16). Subsequent guidelines addressed breast cancer supportive care and survivorship (17, 18), culminating in a landmark endorsement by the American Society of Clinical Oncology (ASCO) (19), which signaled the field’s increasing legitimacy within mainstream oncology.

The collaboration between ASCO and the SIO has since produced comprehensive guidelines addressing symptom-specific management using integrative therapies, including those related to pain (20), anxiety and depression (21), and cancer-related fatigue (22). The evolution of these guidelines reflects both the expansion of the evidence base and the gradual shift from permissive or optional recommendations to more prescriptive guidance when high-quality data support the use of specific modalities. Simultaneously, the National Comprehensive Cancer Network has incorporated integrative recommendations into numerous symptom management pathways, reinforcing the expectation that oncology clinicians should be familiar with evidence-based complementary interventions.

## Clinical application: how integrative oncology works in practice

In clinical settings, integrative oncology is distinguished by its emphasis on personalized, whole-person assessment and support. An integrative oncology consultation typically includes a detailed exploration of lifestyle patterns, psychosocial context, stress and coping strategies, sleep quality, faith or spiritual concerns, social support, the use of herbal and non-herbal dietary supplements or complementary practices, and symptom assessment. Critical to the consultation, depending on the cultural setting, is the proactive and open discussion of which complementary medicine modalities the patient is currently using or considering, given the high prevalence of these practices among individuals undergoing oncology care (23). The integrative oncology consultations are typically adapted across different countries and regions to align with patients’ preferences for and use of diverse integrative modalities. This culturally sensitive approach may acknowledge the widespread use of herbal medicine (24) within traditional systems of medicine such as Traditional Arab and Islamic medicine in the Middle East, Ayurvedic medicine in India, and Traditional Chinese, Korean, and Kampo medicine in East Asia (25), as well as broader health paradigms such as Anthroposophic medicine and homeopathy in European countries. These areas are explored alongside evaluating where patients are in the diagnosis and treatment trajectory, determining comorbidities, and knowing the treatments patients are receiving. This information allows clinicians to construct individualized care plans that are safe and may incorporate nutritional counseling, exercise programs, mind–body interventions, stress reduction strategies, behavioral medicine, and carefully selected complementary therapies delivered by trained practitioners.

This is the approach used by the physicians and practitioners within the Integrative Medicine Center at The University of Texas MD Anderson Cancer Center, Houston, Texas, USA (26, 27) and in the Integrative Oncology Program, Lin-Carmel-Zebulun Medical Centers, Technion-Israel Institute of Technology, Haifa, Israel (28, 29), along with other leading integrative oncology organization around the world (30). This model is not separate from conventional oncology, but rather woven into it, ensuring that supportive interventions align with ongoing treatment and clinical goals, and are patient-family-centered. Other manuscripts in this special issue will go more in-depth with the clinical model and specific integrative oncology approaches and treatments.

## The future: opportunities and challenges

Despite its progress, the field faces persistent challenges. Access to integrative services remains uneven, largely due to inconsistent insurance coverage for evidence-based therapies such as acupuncture, massage therapy, and some forms of behavioral intervention (31). Ensuring equitable access for all patients will require sustained collaboration among clinicians, health economists, policymakers, and insurers. Innovative delivery models, including shared medical appointments, group-based programs, and virtual consultations, may help expand reach while reducing cost and resource burden. These approaches are particularly important in communities where integrative services are limited or where patients face logistical barriers to in-person care. Video-based clinical consultations have been a successful approach to providing more integrative oncology services to a broader audience, including medical consultations, mind-body programming, lifestyle counseling, and more (32). Barriers remain for medical professionals practicing across state lines, but these are surmountable problems if there is a will to create change to support what is best for patients.

Beyond accessibility, equity, and disparities presently challenging the integrative process in clinical practice, integrative oncology should focus more on implementation of clinical guidelines in real-life cancer care. Other future clinical challenges include expansion of the integrative oncology model outside the predominately solid tumor medical oncology setting to hematology oncology, pediatric oncology, surgical oncology, and additional domains. Integrative oncology clinical practice should also be tailored to specific patient populations such as adolescents and young adults (33), patients with cancer experiencing war (34), and patients with diabetes and cancer (35). Additional clinical objectives where integrative oncology can play a role is addressing the burden informal caregivers face while supporting their spouse, parent, child, or friend which often significantly impact their own quality of life (e.g., pediatric oncology setting where parents undergo training in daily relaxation-breathing and manual treatments to enhance their own wellbeing (36)). Above all, integrative oncology is expected to validate the generalizability and ability to implement the clinical model outside the leading, state-of-the-art oncology centers in North America and Europe to more peripheral and culturally diverse populations, while sensitively tailoring the integrative oncology model and process to the patients’ and healthcare provider’s needs, expectations, and resources.

Education remains another critical priority. The future of integrative oncology depends on cultivating a workforce that is proficient in both conventional oncology and evidence-based integrative modalities. Dedicated fellowship programs are essential, as are continuing education opportunities for oncology clinicians seeking greater competency in lifestyle medicine, mind–body interventions, and the safe use of supplements and other natural products. Conversely, practitioners of complementary therapies must be trained in the foundational principles of oncology to ensure patient safety and effective collaboration. Integrating core concepts of whole-person care and evidence-based complementary strategies into medical and nursing curricula would help incorporate these evidence-based approaches and perspectives across the next generation of clinicians. For example, significant steps established so far to address a structured integrative oncology training include an inter-professional consensus process established by members of the SIO (37) and a competency-based medical education (CBME) integrative oncology training program established in Israel on a national scale (38).

Research also needs to continue to expand as integrative oncology becomes more deeply embedded within clinical care. Although the evidence base for many integrative therapies has expanded substantially, important gaps remain, particularly in areas such as spirituality, meaning and purpose, and other existential domains that are central to patient well-being but less amenable to traditional biomedical research approaches. In addition, research should follow a gradual paradigm shift in integrative clinical practice, where the focus on patients’ quality of life improvement should align with the oncologist’s professional needs and gaps in effective conventional treatments (e.g., CIPN, chemobrain, etc.). Integrative oncology research should also consider the medical administrator perspective and examine outcomes such as health resources utilization (e.g., hospital admissions, the use of opioid prescriptions, and cost-effective considerations) (39, 40).

There is the growing need for large-scale pragmatic trials, implementation research, qualitative and narrative-based studies, and whole-systems investigations that evaluate integrative oncology as a comprehensive model of care rather than as a series of isolated therapies. Advances in artificial intelligence, real-world data integration, and precision approaches also provide new opportunities to better understand how behavioral and supportive interventions interact with biological factors, treatment regimens, and patient-reported outcomes. Future integrative oncology research and practice should therefore embrace multi-disciplinary collaboration, bringing together researchers and clinicians from diverse professional backgrounds, while ensuring inclusion of underrepresented voices such as nurses, patient advocates, international representatives, and clinicians with limited research and grant resources. Achieving this vision will require the development of innovative training programs that foster not only “vertical” excellence within specialized research domains, but also a broader “horizontal” understanding of integrative oncology research objectives, methodologies, values, limitations, and potential risks within an inclusive and internationally connected research community.

## Conclusion

Integrative oncology now stands at a promising moment in its development. It has evolved from a peripheral area of interest for health care professionals and patients to a recognized, evidence-informed discipline that meaningfully complements conventional cancer treatment. As clinical programs expand, guidelines mature, and research deepens, integrative oncology is increasingly positioned to influence the broader landscape of cancer care globally. Its central insight, that optimal cancer treatment requires attention to the full spectrum of physical, emotional, social, and existential aspects of health, resonates deeply with the needs of patients and with emerging scientific understanding of the complex interplay between biology, behavior, quality of life, meaning, and clinical outcomes.

The continued evolution of integrative oncology will depend on sustained collaboration among clinicians, researchers, educators, patient advocates, payors, political structures, and society at large. By strengthening the scientific foundation of the field, expanding equitable access to integrative services, and embedding whole-person care principles into the fabric of oncology practice, it is possible to create a future in which cancer care is not only more effective, but also more humane. Ultimately, integrative oncology invites the oncology community to embrace a model of care that acknowledges both the realities of disease and the multidimensional nature of healing; supporting individuals to live with greater resilience, agency, and well-being across the entire cancer and life experience.

## Data Availability

The original contributions presented in the study are included in the article/supplementary material. Further inquiries can be directed to the corresponding author.
